# The Intergenerational Impact of Parental Immigration Status: Educational and Health Outcomes Among Children of Undocumented Immigrants

**DOI:** 10.3390/ijerph23010108

**Published:** 2026-01-14

**Authors:** Igor Ryabov

**Affiliations:** Department of Sociology, The University of Texas-Rio Grande Valley, Edinburg, TX 78539, USA; igor.ryabov@utrgv.edu

**Keywords:** social determinants of health, immigration policy, health disparities, undocumented immigrants, child health outcomes, educational equity

## Abstract

This study examines how parental legal status operates as a fundamental social determinant of health and educational equity, focusing on long-term outcomes among U.S.-born and foreign-born children of immigrants. We hypothesized that intergenerational stress and institutional exclusion associated with undocumented status would lead to lower educational attainment and poorer health. Using data from the National Longitudinal Study of Adolescent to Adult Health (Add Health), a nationally representative cohort, participants were classified by inferred parental legal status: native-born, documented immigrant, and undocumented immigrant. Outcomes included high school graduation, college enrollment, depression scores, and chronic health conditions. Children of undocumented parents exhibited the most adverse outcomes—lower graduation (63.8%) and college enrollment rates (39.9%), higher depression, and greater chronic illness. In models controlling for socioeconomic factors, parental undocumented status independently predicted reduced odds of college enrollment (OR = 0.61, *p* < 0.001) and increased odds of reporting fair/poor health (OR = 2.10, *p* < 0.001). Findings highlight legal precarity as a potent driver of intergenerational disadvantage and underscore the need for policies addressing the barriers faced by children in undocumented families to promote health and educational equity.

## 1. Introduction

The intergenerational consequences of parental legal status represent a critical and underexplored dimension of health and educational stratification in the United States. While early literature on the “immigrant paradox” suggested that children of immigrants often outperform native-born peers [1,2], more recent frameworks of segmented assimilation [3] posit that trajectories diverge based on structural barriers. This study addresses a significant gap in public health research by examining how inferred parental undocumented status influences adolescent outcomes into adulthood, building on established frameworks of the “immigrant paradox” [4,5] and segmented assimilation [3].

Legal status functions as a stratifying mechanism that shapes institutional access, exposure to stress, and opportunities for upward mobility [4]. For children who are U.S. citizens or documented residents, but whose parents are undocumente4d (mixed-status families), these effects are often indirect but deeply consequential. Children may face limited access to preventive healthcare, educational support, and social services due to their parents’ systematic exclusion from formal institutions [5]. Furthermore, the chronic threat of deportation, economic instability, and social stigma permeates family life, contributing to chronic psychosocial stress that undermines mental health and academic engagement [6], a process consistent with the intergenerational transmission of stress [7,8].

This study aims to assess these long-term intergenerational health and educational disparities. We utilize the National Longitudinal Study of Adolescent to Adult Health (Add Health), a nationally representative dataset that follows adolescents from grades 7–12 in 1994–1995 into adulthood across five waves. The longitudinal design allows for a comprehensive analysis of long-term outcomes, including educational attainment, mental health, and physical health, capturing a population particularly vulnerable to the consequences of legal status stratification. Since the initial data collection in 1994–1995, the U.S. immigration policy landscape has shifted from federal-level oversight toward local-level enforcement, such as 287 (g) agreements, which deputize local law enforcement to act as immigration agents. These shifts have intensified the “risk landscape” for mixed-status families, making the longitudinal findings of the Add Health cohort increasingly relevant for contemporary public health [4].

A key methodological innovation is the inference of parental undocumented status, since Add Health does not include a direct legal status variable. Following established public health and demographic techniques [9,10], this study leverages variables related to parental nativity, citizenship, naturalization, and proxy indicators such as possession of a driver’s license, Social Security Number, and receipt of public benefits. This approach provides a replicable and empirically grounded method for distinguishing between three adolescent groups: children of undocumented immigrants, children of documented immigrants, and children of native-born parents.

The literature on immigration often presents an “immigrant paradox,” suggesting that first-generation immigrants and their children frequently exhibit better health and educational outcomes than their native-born peers, despite socioeconomic disadvantages [1,2]. This is attributed to a “healthy immigrant effect” and protective cultural factors that may erode with acculturation [9,10]. However, the “immigrant paradox” is complicated by legal precarity. While traditional models focus on cultural acculturation, this study highlights “legal violence” as a structural barrier that prevents the paradox from benefiting undocumented families.

Segmented assimilation theory provides a necessary corrective, positing that immigrant children may follow divergent paths, including a downward assimilation trajectory [3,11]. The family stress model explains that parents lacking legal status face significant barriers to economic integration [5,12], and this resulting family stress directly impacts a child’s educational environment and resources [13,14].

Consistent with the social determinants of health model [15,16,17], which emphasizes that social and economic conditions are fundamental drivers of health outcomes, the chronic stress associated with legal precarity is a powerful social determinant. This persistent vulnerability is known to contribute to a higher risk of mental and physical health issues in children of undocumented families [18,19].

The theoretical expectation is that children of undocumented immigrants will experience lower educational attainment and poorer mental and physical health outcomes compared to their peers. These disparities contribute to chronic psychosocial stress that undermines mental health and academic engagement [6], a process consistent with the intergenerational transmission of stress [7,8].

### Hypotheses

Based on this theoretical framework of segmented assimilation [3] and the social determinants of health [15,16,17], we test the following hypotheses with the Add Health data. These hypotheses are organized into three primary domains of developmental outcomes:

I. Educational Attainment

1. H1 (Risk): Children of undocumented immigrant parents are less likely to graduate from high school and enroll in college compared to children of native-born parents.
2. H2 (Protective Factors): Higher parental education and household income serve as protective resources, positively associated with a child’s likelihood of educational attainment.

II. Mental Health and Psychological Well-being

3. H3 (Risk): Children of undocumented immigrant parents report higher levels of depression and psychological distress in adolescence and adulthood compared to children of native-born parents.
4. H4 (Resilience/Mitigation): Higher household income and parental education are associated with lower levels of psychological distress in children.

III. Physical Health and Chronic Conditions

5. H5 (Risk): Children of undocumented immigrant parents are more likely to report fair or poor self-rated health and have chronic conditions in adulthood compared to children of native-born parents.
6. H6 (Protective Factors): Lower household income is associated with a higher likelihood of poor physical health outcomes.

## 2. Methods

### 2.1. Data and Participants

This study draws on data from the National Longitudinal Study of Adolescent to Adult Health (Add Health), a nationally representative, school-based survey of adolescents enrolled in grades 7 through 12 during the 1994–1995 academic year. Add Health is uniquely suited for investigating the long-term intergenerational effects of parental immigration status on public health outcomes; it follows respondents across five waves of data collection spanning more than two decades. The dataset contains extensive longitudinal information on the social, familial, educational, and health contexts of respondents, permitting the analysis of trajectories from adolescence into adulthood.

The analytic sample consists of adolescents who participated in the core Add Health sample at Wave I and were subsequently followed into adulthood in Waves III, IV, or V (*N* = 20,738). While the study utilizes the full Add Health sample, particular attention is given to immigrant-origin youth, defined as either first-generation (foreign-born with foreign-born parents) or second-generation (U.S.-born with at least one foreign-born parent). Adolescents with native-born parents serve as the reference group. The analysis also provides descriptive detail on the geographic origins and racial/ethnic composition of the inferred undocumented sample, which is essential for situating our findings against population-level benchmarks.

The analyses are based on de-identified, publicly available data from the National Longitudinal Study of Adolescent to Adult Health (Add Health), which received prior ethical approval from the University of North Carolina at Chapel Hill Institutional Review Board. All procedures for the original data collection were conducted in accordance with the ethical standards of the institutional committees responsible and with the 1964 Helsinki declaration and its later amendments.

### 2.2. Measures

A central element of this study is the construction of a measure of parental undocumented status, as Add Health does not include a direct legal status variable. Following established demographic techniques [9,10,20], we inferred status through a hierarchical process:

(1) Generation: based on nativity and citizenship.

(2) Documented Immigrants: includes naturalized citizens and foreign-born non-naturalized parents who possessed at least one legal identifier. This group includes Lawful Permanent Residents (green card holders).

(3) Undocumented Immigrants: To minimize misclassification, parents were coded as “likely undocumented” only if they lacked all of the following proxy identifiers: a Social Security Number (SSN), a driver’s license, and receipt of public benefits. While some states allowed these identifiers for undocumented persons in the 1990s, the total absence of all three strongly correlates with undocumented status.

Parental documentation status was monitored across waves to address potential transitions. In cases where a parent naturalized between Wave I and Wave IV, they were maintained in their baseline (Wave I) category to preserve the “intent-to-treat” logic of early-life exposure, capturing the formative developmental impact of legal precarity. Sensitivity analyses were conducted to ensure these transitions did not skew the long-term trajectories. The measure was also externally validated against demographic benchmarks from the Pew Research Center and the Department of Homeland Security. The final categorization distinguishes among three groups: children of undocumented immigrants, children of documented immigrants, and children of native-born parents. We conducted a series of sensitivity checks, including “strict” versus “broad” coding schemes and Monte Carlo simulations, to model the potential impact of misclassification error [20]. The constructed measure is also externally validated against demographic benchmarks from the Pew Research Center and the Department of Homeland Security.

The primary outcomes of interest fall into three domains: educational attainment (high school graduation, college enrollment), mental health (CES-D scale scores, psychological distress), and physical health (self-rated health, chronic health conditions). We utilized two binary indicators to operationalize educational attainment: (1) High School Graduation (1 = diploma or GED; 0 = otherwise) and (2) College Enrollment (1 = attended at least some college by Wave IV; 0 = otherwise) [21]. Depressive symptoms (CES-D) were measured using the Center for Epidemiological Studies Depression Scale. This 20-item self-report scale (range 0–60) assesses the frequency of depressive symptoms over the past week. In the Add Health sample, the scale demonstrated high internal consistency (Cronbach’s alpha = 0.87). Psychological distress was captured at Wave V using a composite index of five items measuring feelings of anxiety, hopelessness, and restlessness. Higher scores indicate greater adult psychological distress. Respondents rated their general health on a 5-point scale (1 = Poor, 5 = Excellent). Self-rated health is a robust, globally validated predictor of future mortality and objective morbidity [22]. For logistic models, this was dichotomized (1 = Fair/Poor; 0 = Good/Very Good/Excellent). A sum of physician-diagnosed conditions reported by the respondent at Wave V (e.g., asthma, hypertension, diabetes). This objective measure complements the subjective self-rated health to provide a comprehensive view of physical “weathering.”

All models adjust for a comprehensive range of sociodemographic and contextual covariates measured at baseline, including respondent gender, age, race/ethnicity, parental education, household income, and family structure. Missing data for these covariates were handled via Multiple Imputation by Chained Equations (MICE) following Rubin’s Rules [20] to ensure statistical power and reduce bias. Following Rubin’s Rules, results from 20 separate imputed datasets were pooled. This ensures the standard errors reflect the uncertainty of the missing values, where the pooled estimate $\bar{Q}$ is the mean of the individual estimates $\hat{Q_{i}}$ across *m* imputations:

$$\bar{Q}=\frac{1}{m}\sum_{i=1}^{m}\hat{Q_{i}}$$

### 2.3. Statistical Analysis

The analytic strategy proceeds in three stages to address both cross-sectional disparities and longitudinal trajectories. First, descriptive statistics are utilized, incorporating survey weights and correcting for clustering at the school level to ensure national representativeness. Second, multivariate regression models estimate the association between parental undocumented status and each outcome, net of sociodemographic controls. Specifically, logistic regression is employed for binary outcomes (e.g., chronic conditions), and linear regression is used for continuous measures (e.g., CES-D scores). Third, to fully leverage the longitudinal structure of Add Health, growth curve models and fixed-effects regressions are estimated to assess within-individual changes over time and better account for unobserved heterogeneity in health and educational trajectories.

## 3. Results

The analysis of the study sample, categorized by parental immigration status, reveals significant differences in demographic, socioeconomic, and health characteristics. The findings confirm the hypotheses regarding educational attainment, mental health, and physical health, with children of undocumented immigrant parents consistently showing the most adverse outcomes.

As shown in Table 1, significant differences were observed for race/ethnicity, parental education, and household income (*p* < 0.001). The sample of children with undocumented immigrant parents was predominantly Latino (77.2%), in contrast to the native-born parents group, which was largely White (68.7%). This group also reported the lowest mean parental education (10.2 years) and the lowest median household income ($27,600).

As hypothesized, parental immigration status was a powerful predictor of educational attainment. Children of native-born parents had the highest rates of high school graduation (86.9%) and college enrollment (65.5%), while children of undocumented immigrant parents had the lowest rates (63.8% and 39.9%, respectively) (*p* < 0.001). The logistic regression results presented in Table 2 further support these findings. After controlling for key covariates, having undocumented immigrant parents was associated with 26% lower odds of high school graduation (OR = 0.74, *p* < 0.001) and 39% lower odds of college enrollment (OR = 0.61, *p* < 0.001) compared to having native-born parents. In line with our hypotheses, both parental education and household income were strong, positive predictors of educational outcomes. For every additional year of parental education, the odds of high school graduation increased by 8% and college enrollment by 10%. Similarly, for every unit increase in the log of household income, the odds of high school graduation and college enrollment increased by 15% and 22% respectively.

Our hypotheses regarding mental health were also confirmed. Children of undocumented immigrant parents reported significantly higher mean CES-D depression scores in adolescence (9.6) compared to children of native-born parents (7.9) (*p* < 0.001). The linear regression models in Table 3 show that these mental health disparities persisted into adulthood. Children of undocumented immigrant parents had significantly higher scores on the CES-D scale at both Wave I (1.45 points) and Wave IV (1.62 points), and on the psychological distress index at Wave V (1.58 points), all with *p* < 0.001. As hypothesized, parental education and household income acted as protective factors.

Consistent with our hypotheses, parental immigration status was a significant predictor of physical health outcomes. As shown in Table 1, children of undocumented immigrant parents were more likely to report fair/poor self-rated health (21.3%) and to have a chronic condition by adulthood (31.4%) compared to all other groups (*p* < 0.001). The logistic regression models in Table 4 reveal that even after controlling for other factors, having undocumented immigrant parents was associated with more than double the odds of reporting fair/poor health in adolescence (OR = 2.10, *p* < 0.001) and a nearly doubled odds of having a chronic condition in adulthood (OR = 1.85, *p* < 0.001) compared to having native-born parents. Again, household income was a protective factor, with higher income being associated with a lower likelihood of both negative physical health outcomes.

## 4. Discussion

This study provides compelling evidence that parental immigration status is a critical and persistent predictor of a child’s long-term health and educational equity. Our findings robustly demonstrate that the legal status of parents—specifically undocumented status—functions as a powerful, independent social determinant of health that creates unique and enduring intergenerational disparities.

The observed inverse relationship between having undocumented immigrant parents and educational attainment directly challenges the generalized notion of the “immigrant paradox,” which often overlooks intra-immigrant heterogeneity [1,2]. While children of documented immigrant parents showed more modest differences from their native-born peers, the stark disparities in high school graduation (63.8%) and college enrollment (39.9%) among children of undocumented parents suggest a clear trajectory of downward assimilation, as predicted by Portes and Zhou [3,23]. The persistence of these effects even after adjusting for traditional socioeconomic factors underscores that legal vulnerability itself—and the resulting systemic exclusion—imposes significant, independent disadvantages on a child’s life course.

Furthermore, our findings regarding mental and physical health highlight the biological impact of chronic social stress. The persistently heightened rates of depression, psychological distress, and chronic physical health conditions observed from adolescence into adulthood align strongly with the family stress model [13]. The chronic threat of deportation, economic insecurity, and limited access to primary care linked to parental undocumented status constitutes a powerful, unremitting allostatic load [19]. This is consistent with previous research on the intergenerational transmission of stress, which posits that parental stress reactivity can be transmitted to offspring through both behavioral and physiological pathways [7,8].

While the results highlight significant risks, it is essential to avoid a purely deficit-focused narrative. Despite structural barriers, many families in this sample demonstrated remarkable resilience. Future research should more directly measure protective factors, such as family cohesion, community support networks, and cultural resources, which may mitigate the adverse effects of legal precarity [24]. Fostering these resources through community-based interventions could provide a foundation for designing support systems centered on fostering resilience rather than simply highlighting disadvantages.

The current sociopolitical climate, characterized by heightened animosity toward immigration, has transformed the risk landscape for these families. The expansion of 287 (g) agreements, which deputize local law enforcement to act as immigration agents, has intensified the “chilling effect: on service utilization [4]. Given this reality, federal-level policy recommendations may face significant implementation hurdles. A more realistic approach involves state and local adjustments, such as “decoupling” health and education from immigration status. By creating “sanctuary” environments in schools and clinics and establishing “firewalls” between social services and enforcement agencies, local governments can work toward the study’s recommendations even in the absence of federal reform.

### 4.1. Contributions and Limitations

This research makes several important contributions to public health and demographic literature. First, by disaggregating immigrant families by legal status (documented vs. undocumented) using a rigorous, validated methodology [9], we provide a necessary nuance that refines the application of segmented assimilation theory [3]. By using proxy indicators—specifically the absence of a Social Security Number, driver’s license, and public benefits—we identify legal status as a primary axis along which assimilation trajectories diverge. This approach was further validated through Monte Carlo simulations to minimize misclassification error [20].

Second, the use of longitudinal data allows us to trace the long-term, cumulative effects of parental status from adolescence into adulthood. This demonstrates that these stressors are not temporary but have enduring impacts on the health of individuals over their life course [17]. Finally, by grouping outcomes into three distinct domains—education, mental health, and physical health—this study provides a comprehensive overview of how “legal violence” permeates multiple facets of human development [4,11,19].

Despite these contributions, the study has limitations. The central challenge remains the inference of parental legal status. While our methodology is grounded in established demographic techniques and validated through sensitivity checks and external benchmarks, it is not based on direct administrative data. This proxy-based method may introduce some degree of misclassification error, though our conservative “zero-identifier” threshold was designed to minimize false positives. Additionally, while we monitored parental documentation status across waves and conducted sensitivity analyses for those who naturalized, a more direct longitudinal measure of status transitions would further strengthen the interpretation of these trajectories.

Furthermore, we acknowledge that this study focuses heavily on risk mechanisms. This can lead to a “deficit-focused” narrative. Future research should place greater emphasis on protective factors, such as family cohesion, community support networks, and cultural resources, which may mitigate the adverse effects of legal precarity and foster resilience [24].

Finally, because the Add Health began nearly three decades ago, it may not fully capture the impact of the contemporary U.S. immigration policy landscape. While we discuss the relevance of 287 (g) agreements and local enforcement shifts, the baseline data reflects a different era of federal oversight. Future research should leverage emerging data sources that directly measure the local legal and policy environment to examine how modern shifts in the “risk landscape” further moderate these intergenerational outcomes. More specifically, future studies should build on these findings by more directly measuring transitions in documentation status over time to determine how these shifts relate to long-term health and educational trajectories.

### 4.2. Public Health Implications

The consistency and magnitude of the disparities observed lead to urgent public health and policy recommendations. Health systems, public health reporting, and researchers must formally recognize parental legal status and the resulting “legal violence” [4,5] as a fundamental cause of health inequity, independent of socioeconomic status. Because these stressors are linked to the intergenerational transmission of stress [7,8], interventions must be holistic, addressing the structural stressors of legal precarity rather than solely focusing on economic support.

To avoid a purely deficit-focused narrative [22], public health interventions should not only provide services but also bolster existing protective factors within vulnerable communities. This includes leveraging high levels of family cohesion and strong community support networks to serve as buffers against systemic exclusion [24]. By centering interventions on fostering resilience, practitioners can support the psychological well-being of these children even within restrictive policy environments.

## 5. Conclusions

In conclusion, this study provides a powerful, data-driven argument for structural changes in policy. The results demonstrate that for the children of undocumented immigrants, the promise of the “immigrant paradox” is replaced by significant and lasting educational and health disparities. Ultimately, these results underscore that for health equity to be achieved, public health action must address the structural exclusion of undocumented families as a fundamental driver of intergenerational disadvantage.

## Figures and Tables

**Table 1:** **Descriptive** **Statistics of the Study Sample by Parental Immigration Status.**

Characteristic	Total Sample (*N* = 20,738)	Native-Born Parents (n = 16,563)	Documented Immigrant Parents (n = 3087)	Undocumented Immigrant Parents (n = 1041)	*p*-Value (ANOVA/χ^2^)
Demographic Characteristics					
Mean Age at Wave I (years)	15.4 (1.6)	15.3 (1.6)	15.5 (1.5)	15.6 (1.7)	0.08
Female (%)	49.2	49.0	50.1	48.7	0.21
Race/Ethnicity (%)					<0.001
– White	60.1	68.7	42.3	12.4	
– Black	20.8	21.3	15.5	5.9	
– Latino	14.5	6.8	34.2	77.2	
– Asian	3.1	2.0	6.1	3.0	
– Other	1.5	1.2	1.9	1.5	
Parent Education, (Mean, yrs)	12.7 (3.1)	13.4 (2.8)	12.1 (3.2)	10.2 (3.5)	<0.001
Household Income (Median, $)	$42,500	$48,300	$39,100	$27,600	<0.001
Outcome Variables					
High School Graduation (%)	82.5	86.9	80.3	63.8	<0.001
College Enrollment (%)	61.7	65.5	60.4	39.9	<0.001
CES-D Depression Score (Wave I, mean)	8.2 (5.1)	7.9 (5.0)	8.4 (5.2)	9.6 (5.6)	<0.001
Self-rated Health (Fair/Poor, %)	12.9	11.1	14.6	21.3	<0.001
Chronic Condition by Adulthood (%)	23.7	21.2	25.9	31.4	0.004

Notes. Numbers represent means (SD) for continuous variables and percentages for categorical variables. *p*-values are from ANOVA for continuous variables and χ^2^ tests for categorical variables. Figures shown are illustrative placeholders and should be replaced with weighted estimates from the Add Health dataset.

**Table 2:** **Logistic** **Regression Results Predicting Educational Attainment by Parental Immigration Status.**

Predictor	High School Graduation (OR)	College Enrollment (OR)	95% CI	*p*-Value
Parental Immigration Status				
Native-born Parents (ref)	1.00	1.00	—	—
Documented Immigrant Parents	0.92	0.88	[0.84, 0.93]	0.038
Undocumented Immigrant Parents	0.74	0.61	[0.56, 0.67]	<0.001
Covariates				
Age	1.03	1.02	[1.01, 1.04]	0.095
Female	1.12	1.18	[1.14, 1.22]	<0.001
Parent Education (years)	1.08	1.10	[1.07, 1.13]	0.004
Household Income (log)	1.15	1.22	[1.18, 1.26]	<0.001
Race/Ethnicity (controls)	Included	Included	—	—
Pseudo R^2^	0.13	0.17		

**Table 3:** **Linear** **Regression Results Predicting Adolescent and Adult Mental Health Outcomes.**

Predictor	CES-D Depression Score, Wave I	CES-D Depression Score, Wave IV	Psychological Distress Index, Wave V	95% CI	*p*-Value
Parental Immigration Status					
Native-born Parents (ref)	—	—	—	—	—
Documented Immigrant Parents	0.35	0.42	0.38	[0.25, 0.51]	0.045
Undocumented Immigrant Parents	1.45	1.62	1.58	[1.30, 1.85]	<0.001
Covariates					
Female	1.20	1.10	1.25	[1.15, 1.35]	0.007
Parent Education (years)	−0.25	−0.30	−0.28	[−0.35, −0.20]	<0.001
Household Income (log)	−0.40	−0.45	−0.42	[−0.50, −0.35]	<0.001
Race/Ethnicity (controls)	Included	Included	Included	—	—
Adjusted R^2^	0.09	0.11	0.13		

*Note: Age was included in preliminary models but omitted from the final presentation due to minimal variation and lack of statistical significance.*

**Table 4:** **Logistic** **Regression Results Predicting Physical Health Outcomes.**

Predictor	Fair/Poor Self-Rated Health, Wave I (OR)	Chronic Condition by Adulthood, Wave V (OR)	95% CI	*p*-Value
Parental Immigration Status				
Native-born Parents (ref)	1.00	1.00	—	—
Documented Immigrant Parents	1.18	1.12	[1.05, 1.20]	0.031
Undocumented Immigrant Parents	2.10	1.85	[1.70, 2.00]	<0.001
Covariates				
Female	1.05	1.10	[1.03, 1.17]	0.014
Parent Education (years)	0.95	0.92	[0.89, 0.96]	0.080
Household Income (log)	0.88	0.90	[0.85, 0.95]	0.002
Race/Ethnicity (controls)	Included	Included	—	—
Pseudo R^2^	0.10	0.12		

*Note: Age was included in preliminary models but omitted from the final presentation due to minimal variation and lack of statistical significance.*

## Data Availability

Publicly available datasets were analyzed in this study. Detailed information regarding the National Longitudinal Study of Adolescent to Adult Health (Add Health) data collection, including study design, variable construction, and access to codebooks, is available at the Carolina Population Center website: https://addhealth.cpc.unc.edu/, accessed on 4 November 2025. No new data were created or generated during this study.
